# Chromosome-Level Genome Assembly of the Green Peafowl (*Pavo muticus*)

**DOI:** 10.1093/gbe/evac015

**Published:** 2022-02-02

**Authors:** Xinyuan Zhang, Chuyu Lin, Haimeng Li, Sixia Liu, Qing Wang, Shangchen Yang, Minhui Shi, Sunil Kumar Sahu, Yixin Zhu, Jiangang Wang, Junxuan Huang, Yiyin Hu, Jieyao Yu, Shaofang Zhang, Guanglong Li, Wenyuan Guan, Haorong Lu, Tianming Lan, Yanchun Xu

**Affiliations:** 1 College of Wildlife and Protected Area, Northeast Forestry University, Harbin, China; 2 Shenzhen Zhong Nong Jing Yue Biotech Company Limited, Shenzhen, R & D Center, China; 3 State Key Laboratory of Agricultural Genomics, BGI-Shenzhen, Shenzhen, China; 4 College of Life Sciences, University of Chinese Academy of Sciences, Beijing, China; 5 College of Life Sciences, Zhejiang University, Hangzhou, China; 6 China National GeneBank, BGI-Shenzhen, Shenzhen, China; 7 Guangdong Provincial Key Laboratory of Genome Read and Write, BGI-Shenzhen, Shenzhen, China; 8 China National Forestry Industry Federation, Beijing, China; 9 Quzhou Yueniao Agricultural Development Co. Ltd, Quzhou, China

**Keywords:** green peafowl, chromosome-level genome assembly, PacBio sequencing, conservation

## Abstract

The green peafowl (*Pavo muticus*) is facing a high risk of extinction due to the long-term and widespread threats of poaching and habitat conversion. Here, we present a high-quality chromosome-level genome assembly of the green peafowl with high contiguity and accuracy assembled by PacBio sequencing, DNBSEQ short-read sequencing, and Hi-C sequencing technologies. The final genome size was estimated to be 1.049 Gb, whereas 1.042 Gb of the genome was assigned to 27 pseudochromosomes. The scaffold N50 length was 75.5 Mb with a complete BUSCO score of 97.6%. We identified W and Z chromosomes and validated them by resequencing 14 additional individuals. Totally, 167.04 Mb repetitive elements were identified in the genome, accounting for 15.92% of the total genome size. We predicted 14,935 protein-coding genes, among which 14,931 genes were functionally annotated. This is the most comprehensive and complete de novo assembly of the *Pavo* genus, and it will serve as a valuable resource for future green peafowl ecology, evolution, and conservation studies.

Significance

The improved chromosome-level genome with improved genome annotation of the green peafowl provides opportunities for more reliable and accurate genome-wide analysis, especially for evaluating the genomic consequences of its declining population, furthering our understanding of the ecology, evolution, and conservation of this species.

## Introduction

The green peafowl is one of the most attractive pheasants. Its striking and long feathers are usually regarded as great ornaments, especially the tail feathers (McGowan and Kirwan 2019). It is commonly distributed in East and Southeast Asia (McGowan et al. 1998), but has been experiencing a sharp population decline over the past three decades, largely due to the long-term and widespread threats by human activities such as poaching and habitat conversion (McGowan et al. 1998; Kong et al. 2018). Currently, the population of green peafowl has diminished from most of its historical ranges, and now they are distributed in scattering areas with small and isolated populations (McGowan and Kirwan 2019). This pattern reduces the chance of gene flow and further leads to progressive loss of genetic diversity, which would substantially impair the potential of survival. Due to the high risk of extinction, it is classified as “endangered” in the International Union for the Conservation of Nature (IUCN) Red List (Kong et al. 2018; Wu et al. 2019), thereby urgently requiring systematic conservation efforts.

Genomic analysis is essential for making strategies for the protection and conservation of endangered animals. These analyses provide necessary information of local or meta-populations, such as genetic diversity, gene flow, phylogenetic relationships, genetic loads on genome, inbreeding, and outbreeding effects on individuals or populations, as well as adaptive evolution. A high-quality reference genome at chromosome level will greatly improve the above-mentioned analysis, especially for precise estimation of inbreeding effects by analyzing runs of homozygosity (ROH) and genetic load. Recently, a de novo assembled draft genome of the green peafowl was reported (Dong et al. 2021). However, it was assembled using the second-generation sequencing data only, which is inevitable with high fragmentations and errors (Mittal et al. 2019). Such flaws in quality often lead to bias in the estimation of genetic parameters and genome characterizations.

Therefore, we assembled the first chromosome-level genome of a green peafowl by using the state-of-the-art genome sequencing technologies, comprising Pacific Bioscience (PacBio) long reads, DNBSEQ short reads, and Hi-C sequencing data. We showed obvious improvement in quality, contiguity and accuracy when compared with the previously published genome. This significantly improved assembly will provide a valuable and useful resource for future studies on ecology, evolution, and conservation of this species.

## Results

### Genome Assembly

**Fig. 1.**
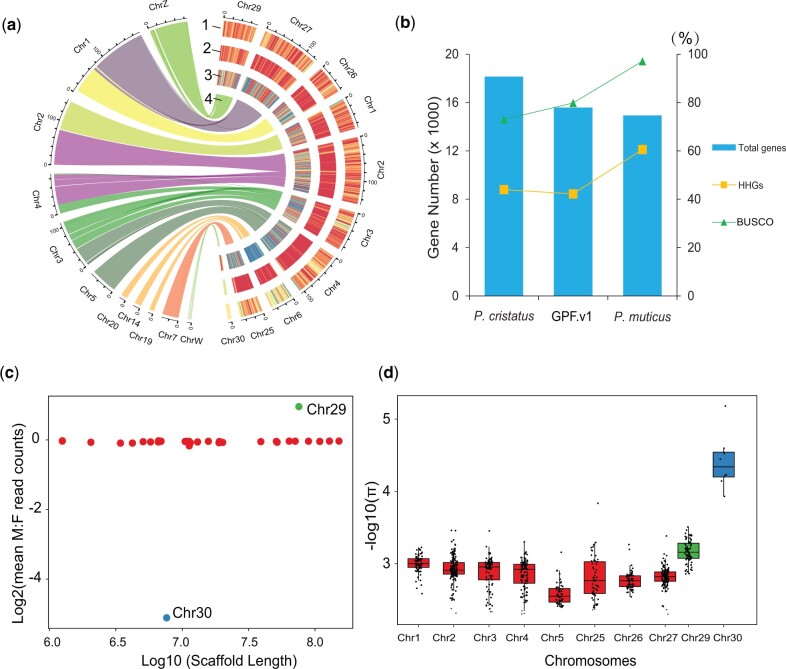
Genome architecture of *Pavo muticus*. (*a*) Genome features and syntenic analysis with the chicken genome. 1: GC content; 2: repeat density; 3: gene density; 4: syntenic result. (*b*) Improved genome annotation. HHGs represented the number of highly homologous genes with chicken’s gene set. (*c*) Averaged male: female depth ratios of scaffolds longer than 1 Mb. Red dots represented autosomes. (*d*) Genome-wide genetic diversity of eight macrochromosomes and two sex chromosomes in female individuals.

The genome size of the green peafowl was estimated to be 1.05 Gb by analyzing the frequency of 17-mers using ∼139.52 Gb DNBSEQ shotgun reads (table 1 and supplementary fig. S1, Supplementary Material online). We first assembled a primary genome by using 159.45 Gb PacBio subreads with contig N50 of 25.4 Mb (table 1 and supplementary table S1, Supplementary Material online). Contigs were then concatenated to the chromosome-level assembly by Hi-C reads. The final genome size was 1.049 Gb, representing 99.9% of the estimated genome size (table 1). The total number of scaffolds in our assembly was 115 and the final scaffold N50 was 75.5 Mb (table 1). In this genome, we identified 27 pseudochromosomes, including 8 macrochromosomes, 17 microchromosomes and 2 sex-linked chromosomes (fig. 1*a* and supplementary fig. S2, Supplementary Material online). The GC content of this genome was 42.1% (fig. 1*a*). The Benchmarking Universal Single-Copy Orthologs (BUSCO) (Simão et al. 2015) analysis showed that 97.6% complete BUSCO genes were recovered (supplementary table S2, Supplementary Material online). In addition, 99.50% and 99.31% of the DNBSEQ shotgun reads and Hi-C reads were mapped to our final assembly, respectively.

**Table 1:** Statistics of Genome Assembly and Annotation of *Pavo muticus*

Item	Category	Number
Sequencing data	PacBio (Gb)	159.45
	Genome survey (Gb)	139.52
	Hi-C (Gb)	692.45
	RNA-seq (Gb)	35.60
Assembly (PacBio)	Estimated genome size (Gb)	1.05
	Assembled genome size (Gb)	1.049
	Contig number	2324
	Contig N50 (Mb)	25.4
	Longest scaffold (Mb)	113.2
Assembly (Hi-C)	Assembled genome size (Gb)	1.049
	Scaffold number	115
	Scaffold N50 (Mb)	75.5
	Longest scaffold (Mb)	151.5
Annotation	GC content (%)	42.1
	Repeat sequences (%)	14.1
	Number of protein-coding genes	14,935
	Number of functionally annotated genes	14,931
	Average gene length (kb)	20.1
	Average exon length (bp)	171.9
	Average intron length (kb)	2.1
	Average exon per gene	9.8

### Genome Annotation

A total of 167.04 Mb (15.92%) genome sequences were identified as repetitive elements (supplementary table S3, Supplementary Material online). The most dominant repeat element was LINEs (109.9 Mb), followed by LTR (46.6 Mb), DNA (15.3 Mb), and SINEs (0.8 Mb). Approximately 13.6 Mb repeat sequences were unknown. A total of 14,935 protein-coding genes were annotated in the current assembly by combining evidence from transcriptome alignment, de novo prediction, and homology-based prediction (supplementary table S4, Supplementary Material online). Protein-coding regions spanned 2.5% (25.9 Mb) of our assembled genome with an average gene size of 20.1 kb. BUSCO analysis showed that the completeness of this gene set is 97.1% (supplementary table S5, Supplementary Material online), and 14,931 (99.97%) genes were functionally annotated.

### Synteny Analysis and Sex Chromosome Identification

We performed the synteny analysis between the green peafowl genome and the chicken (*G. gallus*) genome (fig. 1*a*). High collinearity with clear one-to-one block was found between the two genomes, validating the accuracy of our assembled genome at the chromosome level. We also found fission and fusion events in this comparison. The Chr2 of the green peafowl genome was identified to be the fusion of Chr2 and Chr4 of the chicken genome. Fusion events were also found in the Chr3, Chr4, and Chr6. In contrast, the Chr1 in the chicken genome was split into Chr26 and Chr27 in the green peafowl genome. Fission events were also found in the Chr2, Chr3, and Chr4 of the chicken genome.

We primarily identified that the Chr29 and Chr30 were the Z and W chromosomes of the green peafowl, according to the high similarity with the Z and W chromosomes of the chicken genome. To further validate our inference, we re-sequenced 14 individuals, including 8 female and 6 male individuals. Then, we mapped the whole-genome sequencing reads of these 14 individuals to our assembled genome. As expected, the sequencing depth of the Chr29 and Chr30 in the female individuals were significantly lower than that of autosomes (fig. 1*c* and supplementary table S6, Supplementary Material online). For the male individuals, however, the depths of the Chr29 were nearly the same as the macrochromosomes (fig. 1*c* and supplementary table S6, Supplementary Material online). Further, we calculated the genome-wide diversity (*π*) of ten chromosomes in the small population. We obtained significantly low *π* values on the Chr30 in female individuals (fig. 1*d*). We then concluded that the newly identified Chr29 and Chr30 were the Z and W chromosomes in the green peafowl genome.

## Discussion

Here, we report the first chromosome-level genome of the green peafowl with ten scaffolds totaling 790.8 Mb anchored to eight macrochromosomes and two sexual chromosomes (chromosome Z and chromosome W). The karyotypic study of the blue peafowl (*Pavo cristatus*), the closest relative of the green peafowl, showed eight pairs of macrochromosomes and one pair of sex chromosome (De Boer and Van Bocxstaele 1981). The correspondence between the karyotypic and genomic results indicated the high accuracy of our assembled genome at chromosome level. The GC content of the newly assembled genome was 42.1%, which is very similar to the chicken (42.3%, GRCg6a) and blue peafowl (42.3%, AIIM_Pcri_1.0) genome. In addition, 98.86% DNBSEQ shotgun reads and 98.80% Hi-C reads were mapped to the previously published genome (Dong et al. 2021) (GPF.v1 here after), which was lower than our assembled genome. Surprisingly, the contig N50 and scaffold N50 of our assembled genome were 279-fold and 37-fold longer than that of the GPF.v1 genome. For the gene set we annotated, the BUSCO score was 17.3% higher than that of the GPF.v1 (supplementary table S5, Supplementary Material online). By comparing the gene set of our assembled genome with that of GPF.v1 genome, we found that the number of genes identified in the two genomes was very similar, but much more genes in our genome were supported by homologous genes in the chicken genome, indicating the superiority in the accuracy of our assembled genome (fig. 1*b*). Taken together, our assembled green peafowl genome is not only the most continuous, complete, and accurate de novo assembly of this species, but also the most continuous de novo assembly of the *Pavo* genus by far. With the much-improved genome annotation, our assembled genome will provide a valuable resource for further research works of the green peafowl on ecology, evolution, and conservation.

## Materials and Methods

### Samples and Ethics Statement

One female green peafowl individual from Xinxing breeding base, Liaoning Province, China was selected for genome assembly. Fresh blood sample (1.5 ml) was collected and immediately frozen in liquid nitrogen for 2 h and then transferred to the −80 °C refrigerator for PacBio sequencing, DNBSEQ sequencing and RNA-seq sequencing; 0.5 ml blood sample was collected for a Hi-C library construction. For the crosslinking of the chromatin, the sample was treated with formaldehyde, and then stored at −80 °C for Hi-C sequencing. Feather samples of 14 individuals were collected from Qinhuangdao Wildlife Park for resequencing. All experiments and project designs were approved by the Institutional Review Board on Ethics Committee of BGI (BGI-IRB E21055).

### DNA and RNA Isolation, Library Construction, and Genome Sequencing

Genomic DNA was extracted using a DNA Extraction Kit (TaKaRa, Dalian, China) following the manufacturer’s protocols. The quality and quantity of total DNA were determined with 1% agarose gel electrophoresis and the NanoDrop 2000 spectrophotometer (Thermo Fisher Scientific, MA). TRlzol reagent (Invitrogen) was used for RNA isolation with the manufacturer’s instructions. Agilent 2100 Bioanalyzer system (Agilent) and Qubit 3.0 (Life Technologies) were used for RNA integrity, quantity, and purity evaluation. Approximate 5 μg of high-quality genomic DNA with an average DNA fragment of ∼20 kb was selected for PacBio library construction and sequencing on the PacBio Sequel II platform (Pacbio Biosciences, CA), following the manufacturer’s protocol strictly. Restriction endonuclease *dpn*II was used for Hi-C library preparation. Short-insert-size genomic DNA and cDNA libraries were constructed according to the manufacture’s instruction, and then subject to the DNBSEQ-T1 sequencer (MGI, China) for 100-bp paired-end sequencing.

### Genome Assembly and Assessment

We estimated the size and heterozygosity of the *P. muticus* genome with a *k*-mer frequency-based method (Lander and Waterman 1988). The de novo assembly was built with PacBio long reads, DNBSEQ short reads and Hi-C sequencing data. The initial contigs were assembled by PacBio long reads with the Canu (v2.0) (Koren et al. 2017) pipeline. Subsequently, the NextPolish software (v1.4.0) (Hu et al. 2020) was used to polish the initial assembly with DNBSEQ short reads. Thereafter, we removed redundant sequences in the assembly by purge_dups (v1.2.5) (Guan et al. 2020). Hi-C clean reads were mapped to the initial genome assembly by using Burrows-Wheeler Aligner (BWA, v0.7.17) (Li and Durbin 2010) software with default parameters. Hi-C data quality control was performed by Juicer (v1.5.7) (Durand et al. 2016). 3d-DNA pipeline (v180922) (Durand et al. 2016) was finally used for assigning contigs to the chromosome-level. To assess the genome completeness of the assembly, we first performed the BUSCO (Simão et al. 2015) analysis using the database of vertebrata_odb9. Then, we mapped the DNBSEQ short reads and Hi-C reads to our assembled genome by BWA *mem* with default parameters to calculate the mapping rate.

### Genome Annotation

We used ab initio prediction and homology-based approach to identify the repetitive regions in the genome assembly. RepeatModeler2 (v2.0.1) (Flynn et al. 2020) was used for ab initio prediction of repeats with default parameters. Then, repeats generated by RepeatModeler were merged to the RepBase as known elements. Finally, RepeatMasker (v4.0.5) (Tarailo‐Graovac and Chen 2009) was performed using a conserved BLASTN search in RepBase library (Jurka et al. 2005) to identify and classify transposable elements. We also applied Tandem Repeats Finder (TRF v4.09) (Benson 1999) to identify and locate tandem repeats. Repeats were masked for gene annotation.

We employed an integrative approach including transcriptome alignment, de novo gene prediction, and homology-based predictions to identify protein-coding genes in the *P.muticus* genome with MAKER (v2.31.8) (Campbell et al. 2014). For de novo gene prediction, we used SNAP (v1.0) (Korf 2004), Genescan (v1.0) (Burge and Karlin 1997), glimmerHMM (v3.0.3) (Majoros et al. 2004) and AUGUSTUS (v2.5.5) (Keller et al. 2011) to identify protein-coding genes in the green peafowl genome. For homology-based predictions, protein sequences of *Homo sapiens*, *Taeniopygia guttata*, *Gallus gallus*, and *Meleagris gallopavo* were first downloaded from “Ensemble” 97 release. Then, we used TBLASTN (v2.2.26) (Mount 2007) to align these protein sequences to the *P. muticus* genome with an *E*-value cut-off of 1e-5. Finally, GeneWise (v2.2.0) (Birney et al. 2004) was used to predict gene models. Additionally, the raw RNA-seq reads were filtered by Trimmomatic (v0.30) (Bolger et al. 2014) and assembled into transcripts using Trinity (v2.13.2) (Haas et al. 2013). Transcripts were aligned against our genome assembly by Program to Assemble Spliced Alignments (PASA) (v2.0.2) (Haas et al. 2008) to obtain gene structures. Gene models obtained from the three above-mentioned methods were combined to form a consensus gene set using MAKER (v2.31.8) (Campbell et al. 2014). All protein-coding genes were functionally annotated by aligning against the public protein sequence databases using BLASTP with an *E*-value ≤ 1e − 5.

### Synteny Analysis

The syntenic blocks between the green peafowl and chicken were deﬁned by MCscan (v. 0.8) (Tang et al. 2008) based on core-orthologous gene sets identiﬁed using BLASTp with *e*-value <= 1e-5. The number of genes required to call synteny was larger than 4.

### Variants Calling and Genetic Diversity Calculation

Resequencing data from 14 individuals were aligned to our assembled genome using the BWA *mem* (v0.7.17) (Li and Durbin 2010) with default parameters. Sentieon (Freed et al. 2017) was used for the genomic variant call format (gVCF) calling of each individual with the DNAseq Haplotyper. Sentieon DNAseq GVCFtyper was then used for joint genotyping on 14 gVCF files. We removed indels and performed hard filtering with “QD < 2.0 ‖ FS greater than 60.0 ‖ MQ < 40.0 ‖ MQRankSum < −12.5 ‖ ReadPosRankSum < −8.0 –filter-name snp_filter.” We also filtered multiallelic variants. The genome-wide diversity (*π*) was calculated based on autosomal SNPs using VCFtools (v4.1) (Danecek et al. 2011).

## Supplementary Material

Supplementary data are available at *Genome Biology and Evolution* online.

## Supplementary Material

evac015_Supplementary_Data
