# Supplementary material for: Chromosome-Level Genome Assembly of the Green Peafowl (Pavo muticus)
Source: Genome Biol Evol. 2022 Feb 2;14(2):evac015. doi: 10.1093/gbe/evac015 (PMC8857919; doi:10.1093/gbe/evac015)
Supplement: evac015_Supplementary_Data [file evac015_supplementary_data.docx]

Table S1 Comparison of the assembly statistics among our assembled green peafowl genome, the previously published green peafowl genome and the *Pavo cristatus* genome*.*

| Assembly Level | Parameters | *Pavo cristatus* (This study) | GPF.v1 | *P. cristatus* |
| --- | --- | --- | --- | --- |
| Scaffold | Maximal length (bp) | 151,458,660 | 2,488,982 | 13,419,224 |
|  | N90 (bp) | 11,250,000 | 487,638 | 3595 |
|  | N50 (bp) | 75,521,020 | 2,028,964 | 190,304 |
|  | number>=100bp | 115 | 2446 | 179,332 |
|  | number>=2kb | 114 | 1973 | 22460 |
|  | Ratio of Ns | 0.01% | 1.85% | 0.39% |
|  | Genome size (bp) | 1,049,237,827 | 1,061,110,005 | 1,027,510,962 |
| Contig | Maximal length (bp) | 8,992,229 | 160,453 | 745,077 |
|  | N90 (bp) | 5,468,259 | 14,778 | 922 |
|  | N50 (bp) | 25,396,383 | 91,093 | 6230 |
|  | number>=100bp | 2087 | 35,111 | 399,426 |
|  | number>=2kb | 1801 | 29,156 | 125,291 |
|  | Genome size (bp) | 1,049,075,364 | 1,041,448,921 | 1,023,463,098 |

Table S2 BUSCO Evaluation and comparison of our assembled green peafowl genome, the previously published green peafowl genome and the *P. cristatus* genome*.*

|  | Complete BUSCOs (%) | Complete and single-copy BUSCOs (%) | Complete and duplicated BUSCOs (%) | Fragmented BUSCOs (%) | Missing BUSCOs (%) |  |
| --- | --- | --- | --- | --- | --- | --- |
| *P. cristatus* | 79.70 | 74.60 | 5.10 | 14.30 | 6.00 |  |
| GPF.v1 | 96.70 | 95.90 | 0.80 | 1.30 | 2.00 |  |
| Our assembled genome | 97.60 | 96.90 | 0.70 | 0.80 | 1.60 |  |

Table S3 Statistics of repeats in our assembled genome.

| Type | Length (bp) | % of genome |
| --- | --- | --- |
| Trf | 13,554,793 | 1.291869 |
| Repeatmasker | 124,499,523 | 11.8657 |
| Proteinmask | 78,515,426 | 7.483085 |
| *De novo* | 131,783,576 | 12.55992 |
| Total | 167,045,927 | 15.92068 |

Table S4 Statistics on functional annotation of green peafowl gene set.

|  | Total | Swissprot-Annotated | KEGG-Annotated | TrEMBL-Annotated | Interpro-Annotated | Overall |
| --- | --- | --- | --- | --- | --- | --- |
| Gene Number | 14,935 | 14,675 | 13,674 | 14,931 | 14,412 | 14,931 |
| Percentage (%) | 100 | 98.26 | 91.56 | 99.97 | 96.5 | 99.97 |

Table S5 BUSCO evaluation and comparison of the newly assembled green peafowl gene set, the previously published green peafowl gene set and the *P. cristatus* gene set*.*

|  | Complete BUSCOs (%) | Complete and single-copy BUSCOs (%) | Complete and duplicated BUSCOs (%) | Fragmented BUSCOs (%) | Missing BUSCOs (%) |
| --- | --- | --- | --- | --- | --- |
| *P. cristatus* | 73.00 | 67.90 | 5.10 | 16.60 | 10.40 |
| GPF.v1 | 79.80 | 77.90 | 1.90 | 10.60 | 9.60 |
| *Pavo cristatus* (This study) | 97.10 | 96.20 | 0.90 | 1.80 | 1.10 |

Table S6 Sequencing depth and coverage of sex chromosomes in 15 individuals.

| Sample | Sex | Sequencing Depth  (X) | Sequencing Coverage  (%) | Mapping rate | Z: Coverage  (%) | Z: Depth  (X) | W: Coverage (%) | W: Depth  (X) |
| --- | --- | --- | --- | --- | --- | --- | --- | --- |
| Genome assembly individual | F | 131.20 | 98.98 | 99.50 | 98.61 | 68.24 | 94.60 | 76.96 |
| qhd_01 | F | 37.50 | 98.67 | 98.83 | 98.21 | 18.86 | 91.07 | 24.16 |
| qhd_02 | F | 25.84 | 98.58 | 99.00 | 98.25 | 13.06 | 90.22 | 16.14 |
| zj_02 | F | 38.31 | 98.67 | 98.81 | 98.15 | 18.47 | 93.84 | 31.96 |
| zj_04 | F | 38.63 | 98.63 | 97.71 | 98.11 | 18.91 | 93.55 | 30.11 |
| zj_06 | F | 37.65 | 98.62 | 98.86 | 98.07 | 18.54 | 93.52 | 29.26 |
| zj_08 | F | 34.02 | 98.62 | 98.94 | 98.07 | 16.90 | 93.57 | 25.56 |
| zj_09 | F | 19.91 | 98.46 | 99.50 | 97.98 | 10.30 | 93.54 | 11.91 |
| zj_10 | F | 18.20 | 98.44 | 99.30 | 97.97 | 9.46 | 93.50 | 10.72 |
| yn_01 | M | 23.22 | 97.70 | 96.71 | 98.45 | 22.68 | 8.95 | 0.42 |
| zj_01 | M | 32.27 | 97.71 | 98.16 | 98.23 | 31.14 | 12.01 | 0.69 |
| zj_03 | M | 40.00 | 97.72 | 98.96 | 98.30 | 38.30 | 12.95 | 0.87 |
| zj_05 | M | 36.39 | 97.71 | 98.51 | 98.25 | 34.87 | 12.78 | 0.74 |
| zj_07 | M | 35.12 | 97.69 | 98.92 | 98.23 | 33.36 | 12.24 | 0.82 |
| zj_11 | M | 20.47 | 97.44 | 99.51 | 98.20 | 20.78 | 7.86 | 0.36 |


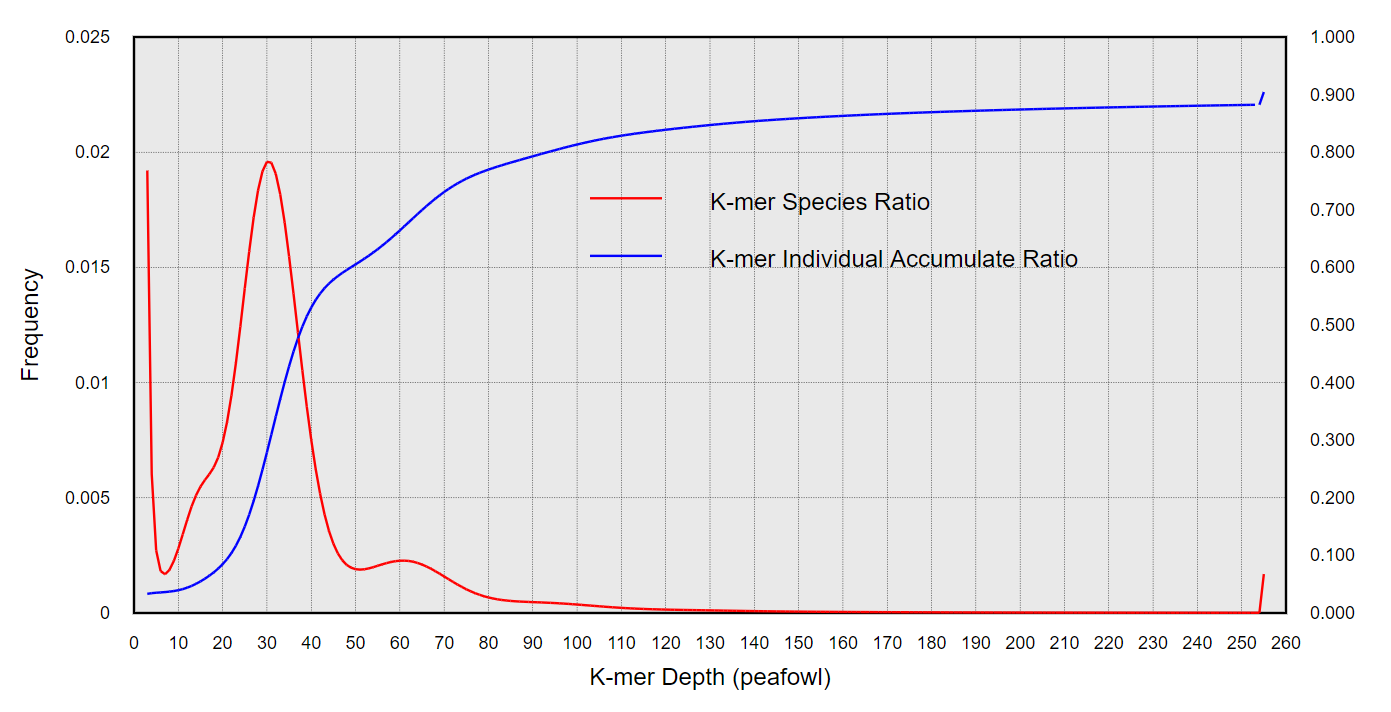


Fig S1. Estimation of the genome size of the green peafowl by K-mer analysis. The red line shows the ratio of k-mer with different depths. The blue line indicates the accumulated ratio of individual k-mers.


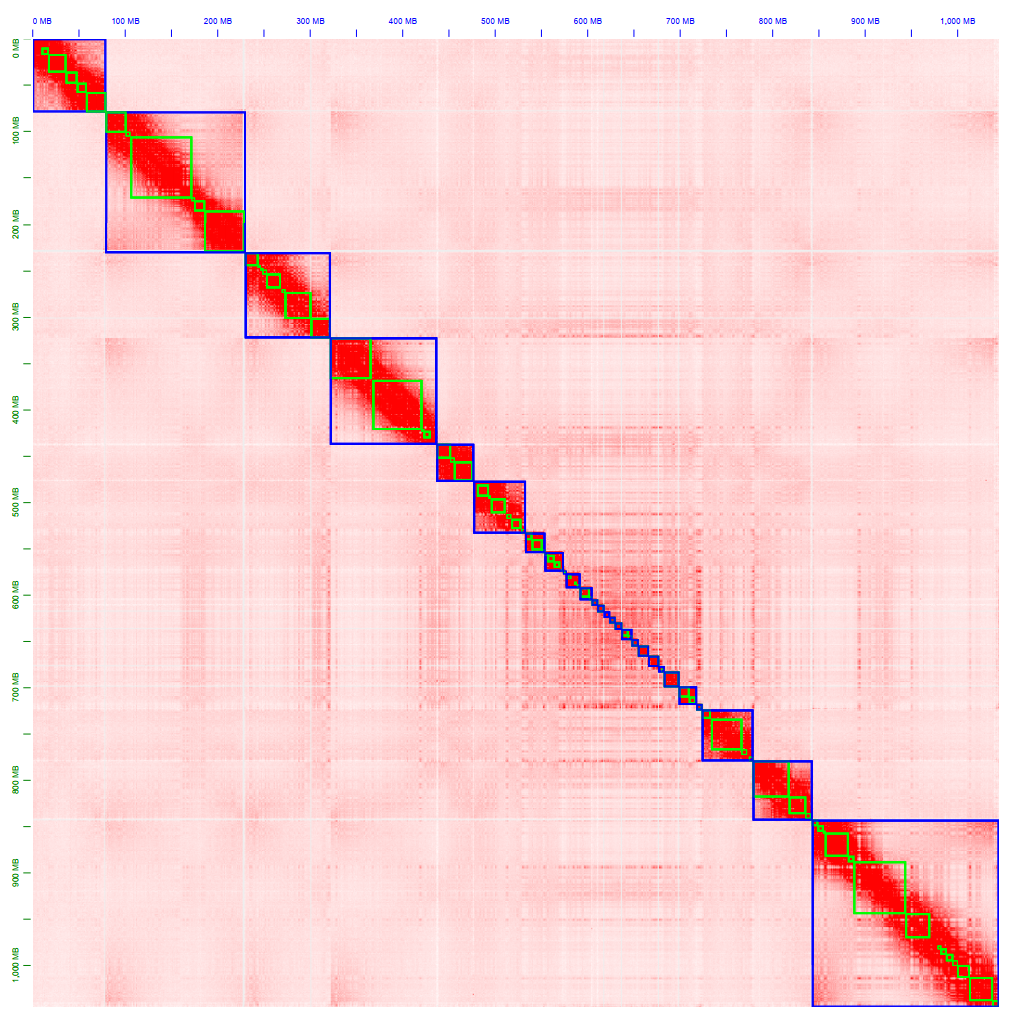


Fig S2. Scaffold contact matrix of the assembled green peafowl genome. The density of Hi-C interactions is represented by the color depth.
